# The MacWilliams Identity for the *m*-Spotty Weight Enumerators over **$Z_{p}R_{k}$**

**DOI:** 10.3390/e28010059

**Published:** 2025-12-31

**Authors:** Juan Wang, An Jiang, Patrick Solé

**Affiliations:** 1 Department of Base Courses, Anhui Sanlian University, Hefei 230601, China; 2 College of General Education, Anhui Wenda University of Information Engineering, Hefei 231201, China; jiang_an@163.com; 3 Lab I2M (CNRS, Centrale Marseille, Aix-Marseille University), 13009 Marseilles, France; sole@enst.fr

**Keywords:** MacWilliams identity, *m*-spotty weight, group character

## Abstract

In this paper, we investigate the *m*-spotty weight enumerators over the mixed alphabet $Z_{p}R_{k}$. Specifically, we construct the Gray map from $Z_{p}^{\alpha }\times R_{k}^{\beta }$ to $Z_{p}^{\alpha +k\beta }$, where $R_{k}=Z_{p}+vZ_{p}+v^{2}Z_{p}+\cdots +v^{k-1}Z_{p}$ with $v^{k}=0$ and k≥5. Based on this framework, we establish the MacWilliams identity for the *m*-spotty weight enumerators between a linear code and its dual over $Z_{p}R_{k}$, by employing the generalized Hadamard transform and the canonical additive character of $Z_{p}$. Finally, an example is presented to illustrate and validate the theoretical results.

## 1. Introduction

In coding theory, it is customary to use generating functions that enumerate codewords by their Hamming weights to evaluate the probability of decoding errors over noisy channels. This generating function, which takes the form of a polynomial, is known as the **weight enumerator**. In 1963, MacWilliams established a fundamental relation between the weight enumerators of a linear code and its dual, now known as the **MacWilliams identity**. This identity has become one of the cornerstones of algebraic coding theory, providing a powerful connection between a code and its dual [1,2,3,4,5].

Error control codes play a vital role in enhancing the reliability of communication and data storage systems. To address more complex noise environments, *spotty* and *m-spotty* byte error models were introduced [6]. These models, which generalize the classical bit-level error model introduced by Shannon, allow for the detection and correction of localized byte errors and thus require generalized weight functions beyond the standard Hamming weight.

In recent years, many researchers have extended the MacWilliams identities to a variety of weights and algebraic structures, including finite rings. For instance, Suzuki et al. [7] derived the MacWilliams identity for binary *m*-spotty weight enumerators. Siap and Özen [8,9] generalized these results to finite fields and to rings such as $F_{2}+uF_{2}$ with $u^{2}=0$, and further to $F_{2}+uF_{2}+\cdots +u^{m-1}F_{2}$ with $u^{m}=0$. They also established the corresponding identities for RT-weight enumerators over arbitrary finite fields [10,11]. Moreover, Siap [12] obtained the MacWilliams identity for *m*-spotty Lee weight enumerators over $Z_{4}$. Subsequently, Sharma and Sharma [12] investigated *m*-spotty weight enumerators over integer modular rings and two-byte error control codes, deriving several applications and their associated MacWilliams identities. A general overview of such developments can be found in [13].

In this work, we continue this line of research by introducing the *m*-spotty weight and the associated weight enumerators over the mixed ring $Z_{p}R_{k}$, where $Z_{p}$ denotes the ring of integers modulo *p* and $R_{k}=Z_{p}+vZ_{p}+v^{2}Z_{p}+\cdots +v^{k-1}Z_{p}$ with $v^{k}=0$ and k≥5. We then establish the MacWilliams identity for linear codes over this mixed alphabet and their duals. The paper is organized as follows. Section 2 recalls basic concepts and preliminaries. Section 3 presents the MacWilliams identity for *m*-spotty weight enumerators over $Z_{p}R_{k}$. Section 4 concludes the paper.

## 2. Preliminaries

Let *R* be a finite commutative ring. A *linear code* of length *n* over *R* is an *R*-submodule of $R^{n}$. A matrix *G* is called a *generator matrix* of the linear code C if the rows of *G* generate C as an *R*-module. For any vector $x=\left(x_{1},x_{2},\ldots ,x_{n}\right)\in R^{n}$, the *Hamming weight* of x is defined as $w_{H}\left(x\right)=|\left\{\;i|x_{i}\neq 0,\;1\leq i\leq n\;\right\}|.$ For any $x=(x_{1},\ldots ,x_{n})$ and $y=\left(y_{1},\ldots ,y_{n}\right)\in R^{n}$, the *Hamming distance* between x and y is

$$d\left(x,y\right)=|\left\{\;i|x_{i}\neq y_{i},\;1\leq i\leq n\;\right\}|.$$

### 2.1. The MacWilliams Identity over the Finite Field $F_{q}$

Let $F_{q}$ denote the finite field with *q* elements, where *q* is a prime power. An [n,k,d] *linear code* C over $F_{q}$ is a *k*-dimensional subspace of $F_{q}^{n}$, where *d* is the minimum nonzero Hamming weight of C. Let $A_{i}$ denote the number of codewords in C with Hamming weight *i*, where i=0,1,…,n. Then the vector $\left(A_{0},A_{1},\ldots ,A_{n}\right)$ is called the *weight distribution* of C, and the polynomial $A\left(z\right)=A_{0}+A_{1}z+\cdots +A_{n}z^{n}$ is the *weight enumerator* of C. The *dual code* of a linear code C is defined by $C^{⊥}=\left\{\;x\in F_{q}^{n}|x\cdot y=0,\;\forall \;y\in C\;\right\}$, where $x\cdot y=x_{1}y_{1}+x_{2}y_{2}+\cdots +x_{n}y_{n}.$ for vectors $x=(x_{1},\ldots ,x_{n})$ and $y=\left(y_{1},\ldots ,y_{n}\right)\in F_{q}^{n}$.

The MacWilliams identity establishes the relation between the weight enumerators of a code and its dual.

**Theorem 1.** 
*Let C be an [n,k] linear code over $F_{q}$ with weight enumerator A(z). Let B(z) denote the weight enumerator of its dual code $C^{⊥}$. Then*

$$B\left(z\right)=q^{-k}{\left(1+\left(q-1\right)z\right)}^{n}A\;\left(\frac{1-z}{1+(q-1)z}\right).$$

### 2.2. Codes over the Mixed Ring $Z_{p}R_{k}$

Throughout this paper, let $Z_{p}$ denote the ring of integers modulo *p*, and let $R_{k}=Z_{p}+vZ_{p}+v^{2}Z_{p}+\cdots +v^{k-1}Z_{p},$ where $v^{k}=0$ and k≥5. For any vectors $x=\left(x_{1},\ldots ,x_{\alpha }|x_{\alpha +1},\ldots ,x_{\alpha +\beta }\right),\;y=\left(y_{1},\ldots ,y_{\alpha }|y_{\alpha +1},\ldots ,y_{\alpha +\beta }\right)$ in $Z_{p}^{\alpha }\times R_{k}^{\beta }$, their *inner product* is defined as

$$\langle x,y\rangle =v^{k-1}\sum_{i=1}^{\alpha }x_{i}y_{i}+\sum_{i=\alpha +1}^{\alpha +\beta }x_{i}y_{i}.$$

For any element $s=s_{0}+s_{1}v+\cdots +s_{k-1}v^{k-1}\in R_{k}$, define the projection $\eta \left(s\right)=s_{0}$. For $s\in R_{k}$ and $x\in Z_{p}^{\alpha }\times R_{k}^{\beta }$, define $sx=(\eta \left(s\right)x_{1},\ldots ,\eta \left(s\right)x_{\alpha }|sx_{\alpha +1},\ldots ,sx_{\alpha +\beta }).$ A linear code C of type (α,β) over $Z_{p}R_{k}$ is defined as a $R_{k}$-submodule of $Z_{p}^{\alpha }\times R_{k}^{\beta }$ under the above multiplication.

The *dual code* of a linear code C of type (α,β) over $Z_{p}R_{k}$ is given by

$$C^{⊥}=\left\{\;y\in Z_{p}^{\alpha }\times R_{k}^{\beta }|\langle x,y\rangle =0,\;\forall \;x\in C\;\right\}.$$

The *Gray map* $\varphi :Z_{p}^{\alpha }\times R_{k}^{\beta }\to Z_{p}^{\alpha +k\beta }$ is defined by

$$\varphi \left(a|b\right)=(a_{1},\ldots ,a_{\alpha }|\phi \left(b_{1}\right),\ldots ,\phi \left(b_{\beta }\right)),$$

where the component map $\phi :R_{k}\to Z_{p}^{k}$ is given by

$$\phi \left(s_{0}+s_{1}v+\cdots +s_{k-1}v^{k-1}\right)=\left(s_{0}+s_{1}+\cdots +s_{k-1},\;s_{1}+\cdots +s_{k-1},\;\ldots ,\;s_{k-1}\right).$$

### 2.3. The m-Spotty Weight over $Z_{p}R_{k}$

Let $c=\left(c_{11},\ldots ,c_{1b},\ldots ,c_{n1},\ldots ,c_{nb}\right)\in R^{nb}$ be a codeword of length N=nb. The *i*-th byte of *c* is denoted by $c_{i}=\left(c_{i1},c_{i2},\ldots ,c_{ib}\right)$. For a byte of length *b*, if at most *t* errors occur in that byte, then such an error is called an *m-spotty byte error* or a *t/b-error*, where 1≤t≤b [14].

**Definition 1.** 
*Let $e\in R^{N}$ be an error vector, and let $e_{i}\in R^{b}$ be its i-th byte (1≤i≤n). Them*-spotty weight *of e is defined as*

$$w_{M}\left(e\right)=\sum_{i=1}^{n}\left\lceil \frac{w_{H}\left(e_{i}\right)}{t}\right\rceil ,$$

*where ⌈x⌉ denotes the smallest integer not less than x [14]. If t=1, then $w_{M}\left(e\right)=w_{H}\left(e\right)$, i.e., the standard Hamming weight. If t=b, then the m-spotty Hamming weight coincides with the usual Hamming weight over $F_{p}^{b}$.*

For $Z_{p}R_{k}=\left\{\left(e_{1},e_{2}\right)|e_{1}\in Z_{p},\;e_{2}\in R_{k}\right\}$, errors may occur in both components $e_{1}$ and $e_{2}$. Since it is difficult to compute the *m*-spotty weight directly on $R_{k}$, we apply the mapping ϕ to convert elements in $R_{k}$ and then compute the *m*-spotty weight by counting the nonzero components.

**Definition 2.** 
*For any element $\left(a^{\alpha }|a^{\beta }\right)=\left(a_{1},\ldots ,a_{\alpha }|a_{\alpha +1},\ldots ,a_{\alpha +\beta }\right)\in Z_{p}^{\alpha }\times R_{k}^{\beta },$ define $w_{M}\left(a^{\alpha }|a^{\beta }\right)=w_{M}\left(a^{\alpha }\right)+w_{M}\left(a^{\beta }\right)$. Let C be a linear code of type (α,β) over $Z_{p}R_{k}$ and N=α+kβ. Then them*-spotty weight enumerator *of C is defined by*

$$W_{M_{C}}\left(x,y\right)=\sum_{c\in C}x^{N-w_{M}\left(c\right)}y^{w_{M}\left(c\right)}=\sum_{i=0}^{N}A_{i}x^{N-i}y^{i},$$

*where $\left(A_{0},A_{1},\ldots ,A_{N}\right)$ denotes the m-spotty weight distribution of C.*

In $Z_{p}$, let $\xi =e^{\frac{2\pi i}{p}}$. The *canonical additive character* of $Z_{p}$ is $\chi \left(x\right)=\xi^{x}$ for $x\in Z_{p}$. Any element $r\in R_{k}$ can be uniquely expressed as

$$r=a_{0}+a_{1}v+\cdots +a_{k-1}v^{k-1},\;a_{i}\in Z_{p}.$$

Then the canonical additive character on $R_{k}$ is defined as

$$\chi \left(r\right)=\xi^{a_{k-1}}.$$

Consequently, for any element $e=\left(e_{1},e_{2}\right)\in Z_{p}R_{k}$, where $e_{1}\in Z_{p}$ and $e_{2}=\sum_{i=0}^{k-1}a_{i}v^{i}\in R_{k}$, the corresponding character is

$$\chi \left(e\right)=\xi^{e_{1}+a_{k-1}}.$$

## 3. MacWilliams Identity for *m*-Spotty Weight over $Z_{p}^{\alpha }\times R_{k}^{\beta }$

In this section, we establish the MacWilliams identity for the *m*-spotty weight over $Z_{p}^{\alpha }\times R_{k}^{\beta }$. Based on Lemmas 2.1, 2.2, and 2.8 in [8], and by following their proof techniques, we derive the following lemmas.

**Lemma 1.** 
*Let H≠{0} be an ideal of $Z_{p}^{\alpha }\times R_{k}^{\beta }$, and let χ be a character over $Z_{p}R_{k}$. Then the sum of the character values of all elements in H equals zero, i.e.,*

$$\sum_{h\in H}\chi \left(h\right)=0.$$

**Lemma 2.** 
*For any $r\in Z_{p}^{\alpha }\times R_{k}^{\beta }$, let χ be a character over $Z_{p}R_{k}$. Then*

$$\sum_{z\in Z_{p}^{\alpha }\times R_{k}^{\beta }}\chi \left(rz\right)=\left\{\begin{array}{cc} |Z_{p}^{\alpha }\times R_{k}^{\beta }|, & r=0,\\ 0, & r\neq 0. \end{array}\right.$$

**Proof.**  There are three cases to consider:

- If r=0, then
  $$\sum_{z\in Z_{p}^{\alpha }\times R_{k}^{\beta }}\chi \left(rz\right)=\sum_{z}\chi \left(0\right)=\sum_{z}1=\left|Z_{p}^{\alpha }\times R_{k}^{\beta }\right|.$$
- If r≠0 and r=v, then by Lemma 1, we have
  $$\sum_{z\in Z_{p}^{\alpha }\times R_{k}^{\beta }}\chi \left(rz\right)=\sum_{z\in \langle v\rangle }\chi \left(z\right)=0,$$
  where 〈v〉 denotes the ideal generated by *v*.
- If r≠0 and r≠v, then similarly, Lemma 1 implies
  $$\sum_{z\in Z_{p}^{\alpha }\times R_{k}^{\beta }}\chi \left(rz\right)=\sum_{z}\chi \left(z\right)=0.$$

This completes the proof. □

**Lemma 3.** 
*Let C be a linear code over $Z_{p}^{\alpha }\times R_{k}^{\beta }$, and let $C^{⊥}$ denote its dual code. Let $f:\;Z_{p}^{\alpha }\times R_{k}^{\beta }\to C$ be a function, and define*

$$\hat{f}\left(e\right)=\sum_{t\in Z_{p}^{\alpha }\times R_{k}^{\beta }}\chi \left(\langle e,t\rangle \right)f\left(t\right),$$

*called the* generalized Hadamard transform *of f(t). Then*

$$\sum_{t\in C^{⊥}}f\left(t\right)=\frac{1}{\left|C\right|}\sum_{e\in C}\hat{f}\left(e\right).$$

**Proof.**  Since $\hat{f}\left(e\right)=\sum_{t\in Z_{p}^{\alpha }\times R_{k}^{\beta }}\chi \left(\langle e,t\rangle \right)f\left(t\right)$, then

$$\begin{array}{cc} \sum_{e\in C}\hat{f}\left(e\right) & =\sum_{e\in C}\sum_{t\in Z_{p}^{\alpha }\times R_{k}^{\beta }}\chi \left(\langle e,t\rangle \right)f\left(t\right)\\ \; & =\sum_{e\in C}\sum_{t\in C^{⊥}}\chi \left(\langle e,t\rangle \right)f\left(t\right)+\sum_{e\in C}\sum_{t\in Z_{p}^{\alpha }\times R_{k}^{\beta }\setminus C^{⊥}}\chi \left(\langle e,t\rangle \right)f\left(t\right). \end{array}$$

For the first half to the right of the equal sign, since $e\in C,t\in C^{⊥}$, then 〈e,t〉=0. By Lemma 2, it is easy to know that the first half is equal to

$$\left|C\right|\sum_{t\in C^{⊥}}f\left(t\right).$$

And for the latter half, since $e\in C,t\in Z_{p}^{\alpha }\times R_{k}^{\beta }\setminus C^{⊥}$, then 〈e,t〉≠0, From Lemma 2, we know that $\sum_{e\in C}\chi \left(\langle e,t\rangle \right)=0$. Therefore

$$\sum_{e\in C}\sum_{t\in Z_{p}^{\alpha }\times R_{k}^{\beta }\setminus C^{⊥}}\chi \left(\langle e,t\rangle \right)f\left(t\right)=\sum_{t\in Z_{p}^{\alpha }\times R_{k}^{\beta }\setminus C^{⊥}}\sum_{e\in C}\chi \left(\langle e,t\rangle \right)f\left(t\right)=0.$$

Hence

$$\sum_{e\in C}\hat{f}\left(e\right)=\sum_{e\in C}\sum_{t\in Z_{p}^{\alpha }\times R_{k}^{\beta }}\chi \left(\langle e,t\rangle \right)f\left(t\right)=\left|C\right|\sum_{t\in C^{⊥}}f\left(t\right),$$

This means that $\sum_{t\in C^{⊥}}f\left(t\right)=\frac{1}{\left|C\right|}\sum_{e\in C}\hat{f}\left(e\right)$. □

**Lemma 4.** 
*Let $u\in Z_{p}^{\alpha }\times R_{k}^{\beta }$ and let χ be a character over $Z_{p}R_{k}$. Then*

$$\sum_{s\in Z_{p}^{\alpha }\times R_{k}^{\beta }}\chi \left(\langle u,s\rangle \right)x^{N-w_{M}\left(s\right)}y^{w_{M}\left(s\right)}={\left(x+\left(p-1\right)y\right)}^{N-w_{M}\left(u\right)}{\left(x-y\right)}^{w_{M}\left(u\right)},$$

*where N=α+kβ.*

**Proof.**  We first define $N=\alpha +k\beta ,u_{\alpha +j}=u_{\alpha +j}^{0}+vu_{\alpha +j}^{1}+\cdots +v^{k-1}u_{\alpha +j}^{k-1}$, $s_{\alpha +j}=s_{\alpha +j}^{0}+vs_{\alpha +j}^{1}+\cdots +v^{k-1}s_{\alpha +j}^{k-1}$, where j=1,2,…,β. Then

$$\begin{array}{cc} \mathrm{left} & =\sum_{s^{\alpha }\in Z_{p}^{\alpha }}\sum_{s^{\beta }\in R_{k}^{\beta }}\chi \left(\langle u^{\alpha },s^{\alpha })x^{\alpha -w_{M}\left(s^{\alpha }\right)}y^{w_{M}\left(s^{\alpha }\right)}\chi \left(\langle u^{\beta },s^{\beta }\rangle \right)x^{k\beta -w_{M}\left(s^{\beta }\right)}y^{w_{M}\left(s^{\beta }\right)}\right.\\ \; & =\left(\prod_{i=1}^{\alpha }\sum_{s_{i}\in Z_{p}}\xi^{u_{i}s_{i}}x^{1-w_{M}\left(s_{i}\right)}y^{w_{M}\left(s_{i}\right)}\right)\left(\prod_{j=1}^{\beta }\sum_{s_{\alpha +j}\in R_{k}}\xi^{u_{\alpha +j}s_{\alpha +j}}x^{k-w_{M}\left(s_{\alpha +j}\right)}y^{w_{M}\left(s_{\alpha +j}\right)}\right). \end{array}$$

For the first half, according to the definition of *m*-spotty weight for any *t*, we have

- If $u_{i}=1$, then $s_{i}\neq 0,w_{M}\left(s_{i}\right)=1$ or $s_{i}=0,w_{M}\left(s_{i}\right)=0$. After substituting these in, we can obtain
  $$\sum_{s_{i}\in Z_{p}}\xi^{0}x^{1-w_{M}\left(s_{i}\right)}y^{w_{M}\left(s_{i}\right)}=\xi^{0}x+\sum_{i=1}^{p-1}\xi^{i}y=x-y.$$
- If $u_{i}=0$, then $s_{i}\neq 0,w_{M}\left(s_{i}\right)=1$ or $s_{i}=0,w_{M}\left(s_{i}\right)=0$. After substituting these in, we can obtain
  $$\sum_{s_{i}\in Z_{p}}\xi^{0}x^{1-w_{M}\left(s_{i}\right)}y^{w_{M}\left(s_{i}\right)}=\xi^{0}x+\left(p-1\right)\xi^{0}y=x+\left(p-1\right)y.$$

Obviously the degrees of x+(p−1)y and x−y are $1-w_{M}\left(u_{i}\right)$ and $w_{M}\left(u_{i}\right)$, respectively. The second half of the proof is similar to the first half, then we have

left=∏i=1α(x+(p−1)y)1−wM(ui)(x−y)wM(ui)∏j=1β∑sα+jl∈Rkl=0,1,…,k−1ξ∑j=1β∑n+l=k−1n,l∈Zpuα+jnsα+jlxk−wM(sα+j)ywM(sα+j)=∏i=1α(x+(p−1)y)1−wM(ui)(x−y)wM(ui)∏j=1β(x+(p−1)y)k−wM(uα+j)(x−y)wM(uα+j)=(x+(p−1)y)N−wM(u)(x−y)wM(u).

This completes the proof. □

**Theorem 2.**  *Let C be a linear code over $Z_{p}^{\alpha }\times R_{k}^{\beta }$. Then the relation between the m-spotty weight enumerator of C and its dual $C^{⊥}$ is*

$$W_{M_{C^{⊥}}}\left(x,y\right)=\frac{1}{\left|C\right|}W_{M_{C}}\left(x+\left(p-1\right)y,x-y\right).$$

**Proof.**  Let $f\left(t\right)=x^{N-w_{M}\left(t\right)}y^{w_{M}\left(t\right)}$, then by Lemma 4 we have

$$\begin{array}{cc} \hat{f}\left(e\right)=\sum_{t\in Z_{p}^{\alpha }\times R_{k}^{\beta }}\chi \left(\langle e,t\rangle \right)f\left(t\right) & =\sum_{t\in Z_{p}^{\alpha }\times R_{k}^{\beta }}\chi \left(\langle e,t\rangle \right)x^{N-w_{M}\left(t\right)}y^{w_{M}\left(t\right)}\\ \; & ={\left(x+\left(p-1\right)y\right)}^{N-w_{M}\left(e\right)}{\left(x-y\right)}^{w_{M}\left(e\right)}. \end{array}$$

According to Lemma 3, for any $e\in C,t\in C^{⊥}$, we have

$$\begin{array}{cc} W_{M_{C^{⊥}}}\left(x,y\right) & =\sum_{t\in C^{⊥}}x^{N-w_{M}\left(t\right)}y^{w_{M}\left(t\right)}\\ \; & =\sum_{t\in C^{⊥}}f\left(t\right)\\ \; & =\frac{1}{\left|C\right|}\sum_{e\in C}\hat{f}\left(e\right)\\ \; & =\frac{1}{\left|C\right|}\sum_{e\in C}\chi \left(\langle e,t\rangle \right){\left(x+\left(p-1\right)y\right)}^{N-w_{M}\left(e\right)}{\left(x-y\right)}^{w_{M}\left(e\right)}\\ \; & =\frac{1}{\left|C\right|}W_{M_{C}}\left(x+\left(p-1\right)y,x-y\right). \end{array}$$

This implies that $W_{M_{C^{⊥}}}\left(x,y\right)=\frac{1}{\left|C\right|}W_{M_{C}}\left(x+\left(p-1\right)y,x-y\right)$. □

Next, we provide a specific example to illustrate our theorem. For convenience, let p=3 and k=2. We consider the MacWilliams identity for the *m*-spotty weight of composite ring $Z_{3}R_{2}=\left\{\left(e_{1},e_{2}\right)|e_{1}\in Z_{3},e_{2}\in R_{2}\right\}$.

**Example 1.** 
*Let C be a linear code over $Z_{3}^{2}\times R_{2}^{2}$ with generation matrix*

$$G_{1}=\left(\begin{array}{cccc} 2 & 0 & v & 0\\ 0 & 1 & 0 & 2v \end{array}\right).$$

*We can verify that the row vector of the matrix is linearly independent and |C|=9. The m-spotty weight for codewords of C can be obtained in Table 1. From this, we can obtain the m-spotty weight distribution of C, as shown in Table 2.*

*Then we can easily calculate the m-spotty weight enumerators of c by the weight distribution $W_{M}\left(c\right)=x^{6}+4x^{4}y^{2}+4x^{2}y^{4}.$ Therefore, by Theorem 2, we can obtain*

$$\begin{array}{cc} W_{M_{C^{⊥}}}\left(x,y\right) & =\frac{1}{\left|C\right|}W_{M_{C}}\left(x+\left(p-1\right)y,x-y\right)\\ \; & =\frac{1}{9}W_{M_{C}}\left(x+\left(p-1\right)y,x-y\right)\\ \; & =\frac{1}{9}\left({\left(x+2y\right)}^{6}+4{\left(x+2y\right)}^{4}{\left(x-y\right)}^{2}+4{\left(x+2y\right)}^{2}{\left(x-y\right)}^{4}\right)\\ \; & =x^{6}+4x^{5}y^{2}+8x^{4}y^{2}+16x^{3}y^{3}+20x^{3}y^{3}+16xy^{5}+16y^{6} \end{array}$$

*This is the m-spotty weight counter for the corresponding dual code $C^{⊥}$.*

## 4. Conclusions

In this paper, we introduced the concepts of error control codes and MacWilliams identities, and defined the *m*-spotty weight over the mixed alphabet $Z_{p}R_{k}$ via a Gray map from $Z_{p}^{\alpha }\times R_{k}^{\beta }$ to $Z_{p}^{\alpha +k\beta }$. By constructing additive characters and employing the generalized Hadamard transform, we derived the MacWilliams identity relating the *m*-spotty weight enumerators of a code and its dual over $Z_{p}R_{k}$. Finally, we provided an explicit example to verify the theoretical results.

## Figures and Tables

**Table 1:** The *m*-spotty weight of codewords of C.

Codeword	$w_{M}\left(c\right)$	Codeword	$w_{M}\left(c\right)$	Codeword	$w_{M}\left(c\right)$
(00|00)	0	(02|0v)	2	(22|vv)	4
(01|02v)	2	(10|2v0)	2	(11|2v2v)	4
(20|v0)	2	(21|v2v)	4	(12|2vv)	4

**Table 2:** Weight distribution of C.

$w_{M}\left(c\right)$	0	1	2	3	4
$A_{i}$	1	0	4	0	4

## Data Availability

No new data were created or analyzed in this study.
